# Detection of insecticide resistance markers in *Anopheles funestus* from the Democratic Republic of the Congo using a targeted amplicon sequencing panel

**DOI:** 10.1038/s41598-023-44457-0

**Published:** 2023-10-13

**Authors:** Holly Acford-Palmer, Monica Campos, Janvier Bandibabone, Sévérin N’Do, Chimanuka Bantuzeko, Bertin Zawadi, Thomas Walker, Jody E. Phelan, Louisa A. Messenger, Taane G. Clark, Susana Campino

**Affiliations:** 1 Department of Infection Biology, Faculty of Infectious and Tropical Diseases, London School of Hygiene and Tropical Medicine, London, UK; 2 Centre de Recherche en Sciences Naturelles de Lwiro, Sud-Kivu, Democratic Republic of the Congo; 3 Médecins Sans Frontières (MSF) OCBA, Barcelona, Spain; 4 Institut de Recherche en Sciences de La Santé (IRSS), Bobo-Dioulasso, Burkina Faso; 5 Université Officielle de Bukavu (UOB), Bukavu, Democratic Republic of the Congo; 6 School of Life Sciences, Gibbet Hill Campus, University of Warwick, Coventry, CV4 7AL UK; 7 Department of Environmental and Occupational Health, School of Public Health, University of Nevada, Las Vegas, Las Vegas, USA; 8 Faculty of Epidemiology and Population Health, London School of Hygiene and Tropical Medicine, London, UK

**Keywords:** Next-generation sequencing, Molecular ecology

## Abstract

Vector control strategies have been successful in reducing the number of malaria cases and deaths globally, but the spread of insecticide resistance represents a significant threat to disease control. Insecticide resistance has been reported across *Anopheles (An.)* vector populations, including species within the *An. funestus* group. These mosquitoes are responsible for intense malaria transmission across sub-Saharan Africa, including in the Democratic Republic of the Congo (DRC), a country contributing > 12% of global malaria infections and mortality events. To support the continuous efficacy of vector control strategies, it is essential to monitor insecticide resistance using molecular surveillance tools. In this study, we developed an amplicon sequencing (“Amp-seq”) approach targeting *An. funestus*, and using multiplex PCR, dual index barcoding, and next-generation sequencing for high throughput and low-cost applications. Using our Amp-seq approach, we screened 80 *An. funestus* field isolates from the DRC across a panel of nine genes with mutations linked to insecticide resistance (*ace-1, CYP6P4, CYP6P9a, GSTe2, vgsc,* and *rdl*) and mosquito speciation (*cox-1, mtND5*, and *ITS2*). Amongst the 18 non-synonymous mutations detected, was N485I, in the *ace-1* gene associated with carbamate resistance. Overall, our panel represents an extendable and much-needed method for the molecular surveillance of insecticide resistance in *An. funestus* populations.

## Introduction

Malaria, caused by *Plasmodium* parasites and transmitted by *Anopheles* spp. mosquitoes, is a major public health problem contributing to substantial global morbidity and mortality^1^. The prevention of malaria relies on vector control measures, particularly the distribution of long-lasting insecticidal nets (LLINs)^1,2^. Whilst LLINs have contributed to significant drops in malaria burden since year 2000, there has been a plateauing in improvements in case reductions, coinciding with the spread of insecticide resistance across many *Anopheles* spp.^3,4^.

Species of the *Anopheles gambiae* (*An. gambiae)* group are the dominant malaria vectors across most of sub-Saharan Africa, but other species from the *An. funestus* group (*An. funest*us sensu stricto, *An. parensis*, *An. vandeeni*, and *An. rivulorum*) are also vectors and contribute to malaria transmission^5–7^. *An. funestus s.s.* mosquitos make up the largest population in the complex and have the widest geographical distribution^8^. This vector can thrive in varying climate conditions, is highly anthropophilic, and has night-time biting and endophilic resting behaviour^3,9^. These behaviours make *An. funestus* highly susceptible to traditional vector control methods, but resistance to insecticides has emerged^3,10–12^.

The Democratic Republic of Congo (DRC) is a malaria hotspot, with > 25 million cases (12% of global total), and transmission caused by both *An. gambiae* and *An. funestus* complex species^1^. Since 2015, there has been a 15% increase in malaria cases in DRC^1,13^, with in-country vector control relying on mass distribution of LLINs, complemented by smaller-scale indoor and outdoors (ORS) residual spraying (IRS) in focal areas, by private mining enterprises. However, resistance to the four major classes of insecticides (carbamates, cyclodienes, organophosphates, pyrethroids) has emerged in *An. gambiae*, and *An. funestus s.s.*^13,14^.

The underlying mechanisms of insecticide resistance across several mosquito species include target site mutations and metabolic-based, but alterations in microbiome composition and cuticles, as well as behavioural modifications, have been found to alter vector susceptibility^15–18^. Target site resistance results from single nucleotide polymorphisms (SNPs) that cause changes to the amino acid sequence in proteins involved in insecticide binding. The most well-known are the *kdr* (knock-down resistance) mutations in the *voltage-gated sodium channel* (*vgsc*), including *kdr* L1014F/S, V410L, F1508C, N1549Y, and D1763Y^19–22^, which result in resistance to pyrethroids and DDT^23,24^. None of these mutations have been described in *An. funestus* populations despite extensive studies^9,25–28^. Other commonly observed mutations in *Anopheles* spp., including the G119S mutation in the *acetylcholinesterase-1* (*ace-1*) gene, leading to resistance to organophosphates and carbamates, but has not been observed in *An. funestus* populations^29–31^. However, the *ace-1* N485I mutation was identified in *An. funestus* samples from Malawi and linked to bendiocarb (carbamate) resistance^32^. Similarly, the A296S mutation in the *gaba* receptor, also known as the *rdl* (resistant to dieldrin) mutation, has been observed in *An. funestus* populationsand linked to several insecticides, including cyclodienes, a subgroup of organochlorides^31,33,34^.

Metabolically mediated resistance mutations include the L119F mutation in the glutathione-S-transferase epsilon 2 (*GSTe2*) gene, which is linked to DDT resistance, and has been found in *An. funestus*^35,36^. Other work in this vector has sought to identify resistance associated alleles in cytochrome P450 genes (e.g., *CYP6P9a* and C*YP6P4*), with pyrethroid resistance linked to overexpression of the *CYP6P9a* gene in isolates from Southern Africa, driven by cis-regulatory polymorphisms^32,37–39^.

The increasing resistance to insecticides in *An. funestus* highlights the need for rapid molecular surveillance techniques to identify underlying mutations, and thereby inform National Malaria Control Program for appropriate decisions about insecticide usage. Whole genome sequencing is limited by a need for high DNA concentrations and the large size of mosquito genomes (~ 350 Mbp) results in a high cost per sample. Amplicon sequencing (“amp-seq”), which can simultaneously target many genomic regions (each ~ 500 bp) across candidate genes, has previously been applied to other vectors such as *An. gambiae, An. stephensi* and *Aedes aegypti*^40–43^. Amplicon primers designed for *An. gambiae* were tested in silico to check whether they were suitable for use on *An. funestus*, however the number of mismatches (n ≥ 4) per primer, when compared to the reference sequence, meant they were unlikely to work efficiently on field specimens. Here we developed a targeted *An. funestus* amp-seq assay and applied it to 80 wild caught mosquitoes from the DRC to screen for molecular markers of insecticide resistance. The 17-amplicon panel covers regions in *vgsc, ace-1*, *CY9P6a*, *CYP9P4*, *GSTe2* and *rdl* loci for insecticide resistance profiling, as well as mitochondrial genes (cytochrome oxidase 1 (*cox-1*), NADH dehydrogenase 5 (*mt-ND5*)) and the ribosomal locus *ITS2* (internally transcribed spacer 2) for speciation and phylogenetic analysis. The amp-seq assay uses a dual index barcoding system to facilitate the pooling of amplicons across many samples, thereby increasing throughput and decreasing costs. Our assay represents a promising strategy to support *An. funestus* vector control surveillance.

## Materials and methods

### Sample collection

Adult *Anopheles* were collected from households in two sites in Sud-Kivu province (Tchonka; 2° 19′ 18″ S, 27° 32′ 24″ E and Tushunguti; 1° 48′ 19″ S, 28° 45′ 00.5″ E) using Centers for Disease Control (CDC) light traps during the rainy seasons (Tchonka: April–June 2018; Tushunguti December 2017–February 2018). Mosquitoes were identified morphologically as members of the *An. funestus s.l.* group^44^. A total of 80 isolates were used for this study (Tchonka 70; Tushunguti 10). Individual mosquitoes were homogenized in a Qiagen TissueLyser II with sterilized 5 mm stainless steel beads for 5 min at 30 Hz and incubated overnight at 56 °C. DNA was extracted using a Qiagen DNeasy 96 blood and tissue kit (Qiagen, UK), according to the manufacturer’s protocol.

### Primer design

Amplicon primers were designed with Primer3 software, using sequences from the *An. funestus* FUMOZ reference strain downloaded from VectorBase^45,46^. The primers were designed to amplify a region of around 500bp, typically around a SNP previously reported as associated with insecticide resistance in *Anopheles, Aedes*, or *Culex* mosquitoes. Where possible, these primers were designed to bind to exons. This panel comprised of 17 primer pairs (amplicons) targeting nine genes, including *vgsc* (6 amplicons), g*aba* (2), *ace-1* (3), *GSTe2* (1), *CYP6P4* (1), *CYP6P9a* (1) for insecticide resistance, and the *cox1* (1 amplicon), *ITS2* (1), and *mt-ND5* (1) for species identification or phylogenetic analysis (Supplementary Table 1). Primers for the CYP6P9a amplicon were taken from Weedall et al.^39^. Each primer was concatenated with one of ten unique 8bp barcodes at the 5′ end. Each sample was assigned a barcode combination to be used throughout amplicon generation. This allowed for amplicons from samples with different barcodes to be pooled. To assess their suitability for multiplexing, samples were checked for potential dimer formation using ThermoFisher Scientific Multiple Primer Analyser software with sensitivity set to one.

### Amplicon generation

Using NEB Q5 hot start polymerase (New England BioLabs, UK), amplicons (500 bp) were generated in 25 μl reactions. Sample volume of 1 μl (~ 2 ng/μl) was used, with an average final primer concentration of 0.5 μM in each PCR. The amplification was conducted as follows: hot-start polymerase activation for 3 min at 95 °C, followed by 30 cycles of 95 °C for 10 s, 60 °C for 30 s and 72 °C for 45 s, followed by a final elongation step of 72 °C for 2 min. Post-multiplex PCR reaction, amplicons were visualised on a 1% agarose gel to confirm band size. Multiplexed PCR amplicons were first pooled by sample, and then with other samples with different 5ʹ barcode combinations. Sample pools were purified using Roche Kapa beads following manufacturer’s instructions. A bead to sample ratio of 0.7:1 was used to remove excess primers and PCR reagents. The Qubit 2.0 fluorimeter HS DNA kit was used to quantify the pool concentration. Illumina adaptors and barcodes were ligated to the sample pool as a part of the Illumina-based Amplicon-EZ service (Genewiz, UK). The indexed pool was then sequenced using a 2 × 250 bp paired end configuration on an Illumina MiSeq. A minimum of 50,000 reads were attained per pool, equating to at least 290 reads per amplicon in a pool of 170 amplicons (at a low cost of < US$0.5 per amplicon).

### Amplicon analysis

The multi-sample fastq files were first demultiplexed using an in-house python script (https://github.com/LSHTMPathogenSeqLab/amplicon-seq) into individual sample fastq files, through the unique barcode combination previously assigned. The reads were trimmed using the Trimmomatic package, then mapped to the reference sequence with bwa-mem and mapped reads clipped using the Samclip package^47–49^. Using the alignments, GATK HaplotypeCaller (v4.1.4.1, default parameters) and Freebayes (v1.3.5, –haplotype-length -1) software were applied for variant calling^50,51^. Any identified SNPs or insertions/deletions (INDELS) were filtered using bcftools for a minimum allele depth of 20 reads. The Phred score was also used for filtering, where a score of > 30 per base was required to pass quality control checks. To determine the consequence of variants at an amino acid level, the SnpEff tool was applied with a database built from the FUMOZ reference genome^52^. The available reference genomes, at the time of analysis, either had no information about insecticide susceptibility, or were the pyrethroid resistant FUMOZ strain. Variants were genotyped using the proportion of alternate allele reads to total position reads for each sample. Samples were genotyped as homozygous reference (< 20% alternate allele), heterozygous (20–80% alternate allele) or homozygous alternate (> 80% alternate allele)^40,41,43^.

### Phylogenetic analysis

For the *ITS2, cox1* and *mt-ND5* amplicons, each sample bam file was converted into fasta format using an in-house script (https://github.com/LSHTMPathogenSeqLab/fastq2matrix). This analysis required a depth of at least 20-fold in each position, and if samples had a large proportion (> 90%) of uncalled bases, they were excluded from this analysis. For each gene, sequences were aligned using MAFFT software, along with publicly available sequences of *An. funestus* specimens from other countries^53^. For *ITS2*, this included 35 samples from Cameroon, DRC, Ethiopia, Kenya, Madagascar, Malawi, Mozambique, and Zambia. The *cox1* alignment included 111 sequences from Cameroon, Central African Republic, DRC, Gabon, Ghana, Kenya, Madagascar, Malawi, Mozambique, Tanzania, Uganda, and Zambia. For mt-ND5, 66 sequences from DRC, Ghana, Kenya, Malawi, Mozambique, Tanzania, Uganda, and Zambia were used. The alignments were then viewed and trimmed in Aliview^54^. RAxML software was used to construct maximum likelihood phylogenetic trees, with a bootstrap value of 1000, and gamma model of heterogeneity and GTR model of nucleotide substitution assumed^55^. The resulting tree model was visualised using iTOL software^56^.

### Haplotype analysis

Specimen sequences were aligned using MAFFT software, and haplotype networks were constructed using the R package Pegas^57^. Amplicon nucleotide diversity and haplotype diversity were also calculated using the Pegas package. The vcftools package was used to calculate nucleotide diversity per SNP, fixation index, and linkage disequilibrium metrics. Linkage disequilibrium output was visualised with the Gaston R package^58,59^.

## Results

### Detection of SNP associated with insecticide resistance

Eighty *An. funestus* specimens were sequenced resulting in the identification of 377 variants (351 SNPs and 26 INDELs not previously described) across the 17 amplicons (Supplementary Table 2). The average coverage of amplicons varied from 193- to 3684-fold. Of the 351 SNPs identified, 92% were either intronic variants or synonymous variants. A total of 18 missense SNPs were found, but no INDELs resulted in amino acid changes (Table 1). Of these 18 non-synonymous SNPs, only one had been previously reported—the N643I mutation in the *ace-1* gene (N485I in *Torpedo californica* otherwise known as Pacific electric ray). The *ace-1* N643I SNP occurred in samples from both Tchonka (7/70) and Tushunguti (1/10). The remaining 17 novel missense SNPs appeared in either the *CYP6P4* (I288N, G289R, N291S/T, L294V, K295E, E297K, D404N, and I414L)*, GSTe2* (G80A, V134M, and K146T) or *vgsc* gene (domain II) (F763L, I768L/M, L788F, and G793C). All SNPs were detected at low frequencies, with allelic frequencies varying from 0.7 to 13.6%. Also identified was a 2bp insertion in the *CYP6P9a* amplicon, which occurs in a non-coding region, thereby not resulting in an amino acid change, but has been identified previously as a marker for pyrethroid resistance^39^. This insertion occurred in 91.3% of samples, with 73.9% of specimen’s genotyped as homozygous alternate (R/R), 17.4% as heterozygous (R/S), and 8.7% as homozygous reference (S/S).

**Table 1:** Location and frequencies of non-synonymous variants detected.

Amplicon	Position	Sample number	Annotation	Allele frequencies		Nucleotide diversity
				Reference	Non-reference	
ACE1III	19,555,221	68	Asn643Ile	91.9	8.1	0.25
CYP6P4	8,560,733	66	Ile414Leu	97.7	2.3	0.07
	8,560,763	66	Asp404Asn	93.2	6.8	0.16
	8,561,152	66	Glu297Lys	92.4	7.6	0.19
	8,561,158	66	Lys295Glu	92.4	7.6	0.19
	8,561,161	66	Leu294Val	96.2	3.8	0.14
	8,561,169	66	Asn291Ser	97.8	2.2	0.07
	8,561,169	66	Asn291Thr	94.7	5.3	0.14
	8,561,176	66	Gly289Arg	98.5	1.5	0.06
	8,561,178	66	Ile288Asn	91.7	8.3	0.18
GSTe2	75,252,570	70	Lys146Thr	93.6	6.4	0.18
	75,252,607	70	Val134Met	99.3	0.7	0.03
	75,252,839	70	Gly80Ala	86.4	13.6	0.27
VGSCIIa	42,339,660	71	Phe763Leu	99.3	0.7	0.14
	42,339,675	71	Ile768Leu	97.9	2.1	0.11
	42,339,677	71	Ile768Met	98.9	1.4	0.14
	42,339,735	71	Leu788Phe	97.2	2.8	0.15
	42,339,750	71	Gly793Cys	97.9	2.1	0.11

Linkage disequilibrium was calculated for each *CYP6P4* and *vgsc* (domain II) amplicons due to the high number of non-synonymous SNPs present. For the VGSCIIa amplicon, perfect linkage disequilibrium (LD r^2^ = 1) was present between the I768L and G793C mutations. High LD was observed in the VGSCIIa amplicons between other sets of SNP pairs (F763L, I768M; F763L, L788F; I768M, L788F; I768L, G793C; all r^2^ > 0.75), suggesting a strong association between these mutations. In the *CYP6P4* amplicons, perfect linkage (r^2^ = 1) was observed between E297K and K295E, and L294V and N291T SNPs (Supplementary Fig. 1).

### Genetic diversity of *An. funestus* in Eastern DRC

**Figure 1:**
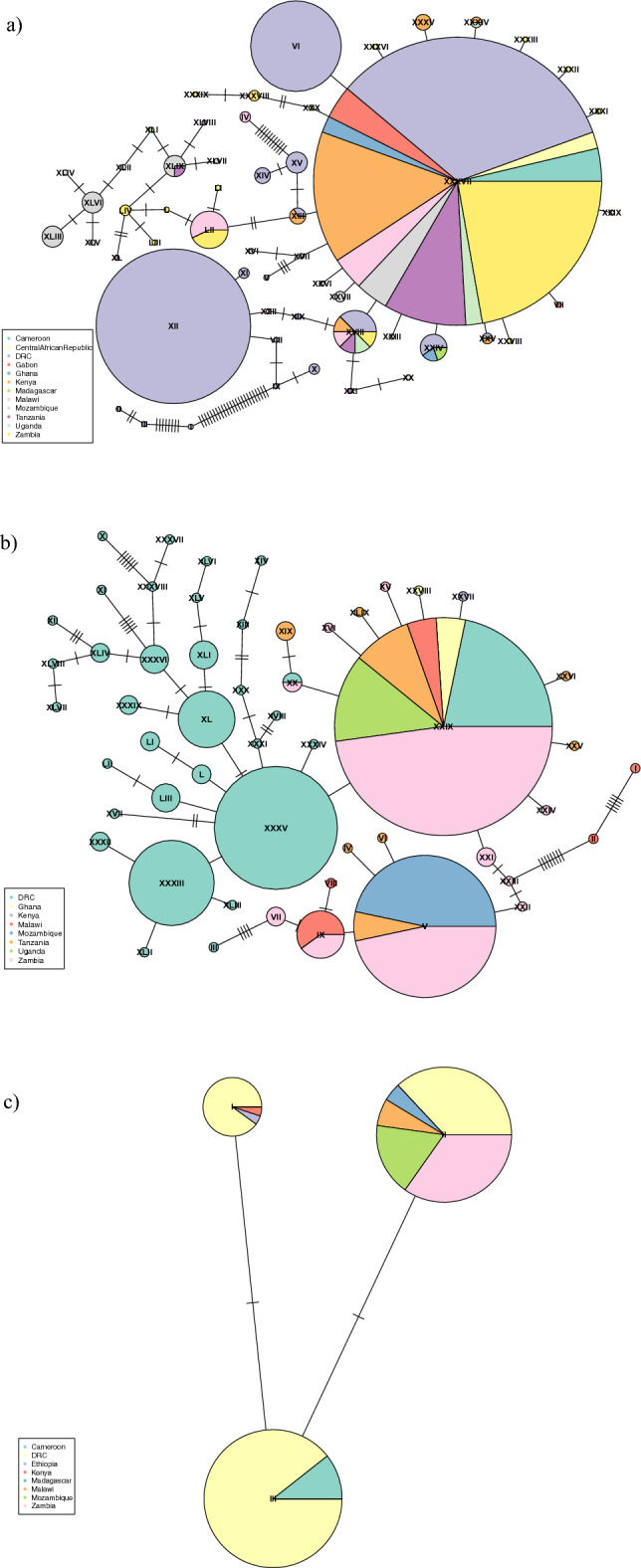
Haplotype or minimal-spanning network constructed using (**a**) *cox-1*, (**b**) *mt-ND5,* and (**c**) ITS2 sequences generated in this study and publicly available samples. Each node represents a haplotype, each segment within the node represents a country, and is proportionally sized to the number of sequences present in the segment and node. The number of ticks between nodes represents the number of genetic differences between nodes.

Low genetic diversity was observed at all three loci (Table 2). The *cox-1* gene had the highest nucleotide diversity (0.011) and number of SNPs identified (64/351). This high number of SNPs resulted in high haplotype diversity, with 18 haplotypes identified and 50% being singletons. When 111 *cox-1* sequences from 11 other countries were included, 54 haplotypes were identified, 32 (58%) of which were singletons. The *mt-ND5* gene also exhibited high haplotype diversity, but from a smaller number of SNPs (n = 13), with 17 haplotypes identified (53% singletons). The number of haplotypes identified expanded to 53 (66% singleton) when including in the analysis 66 publicly available *mt-ND5* sequences covering seven countries. For *ITS2* sequences from DRC*,* four SNPs were identified, resulting in three haplotypes none of which had fewer than seven isolates present. When expanding these networks to include publicly available *ITS2* sequences (n = 35; 8 countries), the number of haplotypes remained the same (n = 3), with > 20 isolates per haplotype. The haplotype networks for each gene (Fig. 1a–c), showed that most samples from the different countries shared a core haplotype, including the DRC samples. For the *ND5* sequences many DRC samples had haplotypes that were not present in the other countries (Fig. 1b).

**Table 2:** Haplotype and nucleotide diversity of genes in DRC.

Gene	No. of haplotypes	Haplotype diversity	Nucleotide diversity
ITS2	3	0.60	0.002
Cox1	18	0.82	0.011
ND5	16	0.93	0.006

**Figure 2:**
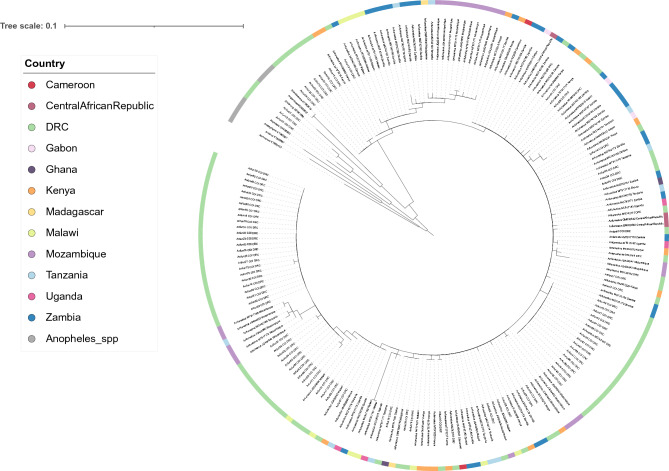
Maximum-likelihood tree constructed using *cox-1* gene sequences generated in this study (n = 84), alongside other publicly available *An. funestus cox-1* sequences (n = 111), (Cameroon = 2, Central African Republic = 3, DRC = 7, Gabon = 3, Ghana = 2, Kenya = 16, Madagascar = 2, Malawi = 11, Mozambique = 21, Tanzania = 10, Uganda = 4, Zambia = 30). This tree also has a group of *Anopheles* spp. (n = 7), including *An. arabiensis, An. darlingi, An. dirus, An. gambiae s.s, An. minimus, An. sinensis* and *An. stephensi.* The tree was built using the maximum-likelihood method assuming GTR model of nucleotide substitution, with the gamma model of heterogeneity rate.

**Figure 3:**
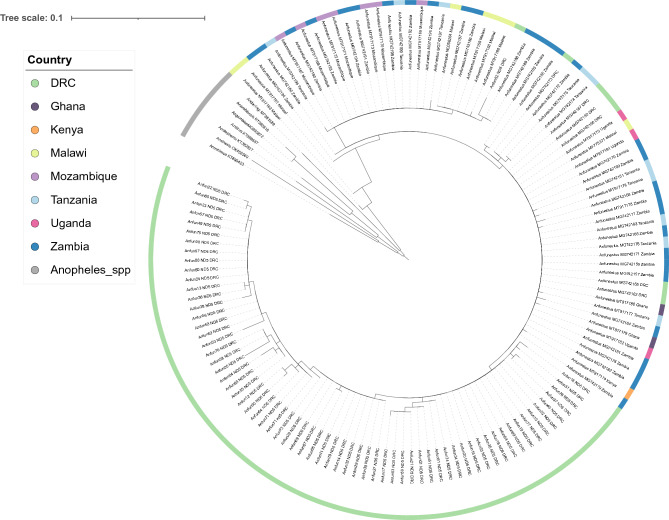
Maximum-likelihood tree constructed using *mt-ND5* gene sequences generated in this study (n = 67), alongside other publicly available *An. funestus mt-ND5* sequences (n = 66), (DRC = 6, Ghana = 2, Kenya = 1, Malawi = 7, Mozambique = 7, Tanzania = 10, Uganda = 3, Zambia = 30). This tree also has a group of *Anopheles* spp. (n = 7), including *An. arabiensis, An. darlingi, An. dirus, An. gambiae s.s, An. minimus, An. sinensis* and *An. stephensi.* The tree was built using the maximum-likelihood method assuming GTR model of nucleotide substitution, with the gamma model of heterogeneity rate.

The data from the three genes demonstrated little population differentiation within the phylogenetic tree constructed (Figs. 2, 3; Supplementary Fig. 2). Both mitochondrial genes demonstrated an ability to speciate, with separate clades for each *Anopheles* spp. (Figs. 2, 3). The *cox-1* gene was able to distinguish at a greater resolution, with samples likely to be incorrectly identified through morphology as *An. funestus* (Anfun01, Anfun13, Anfun27 and Anfun71) appearing within the other *Anopheles* spp. clade. Anfun71 was in a cluster with the *An. arabiensis* and *An. gambiae s.s.* sequences, which was supported by a NCBI BLAST analysis that revealed it shares a 99.0% identity with *An. gambiae cox-1* isolates, and a 98.5% identity with *An. arabiensis cox-1* sequences. NCBI BLAST identified the remaining three samples (Anfun01, Anfun13, Anfun27) as *An. coustani* (identity > 97%). In comparison, the *mt-ND5* gene did not speciate these samples as non-*An. funestus* but did reveal the clearest population differentiation between the DRC isolates and the publicly available sequences from other countries (Fig. 3).

## Discussion

The application of our amp-seq panel to *An. funestus* collected in Eastern DRC demonstrates its utility as a surveillance technique for genotypic-based insecticide resistance and species identification. Across the 80 DRC samples, we identified the *ace-1* N643I SNP, alongside 17 other putatively novel non-synonymous SNPs in genes associated with insecticide susceptibility. The *ace-1* N643I resistant allele, also known as N485I in *Torpedo californica*, was originally found in Southern African countries such as Mozambique and Malawi, but appears to have spread or emerged independently in DRC^32^. This SNP has been associated with increased resistance to the carbamate, bendiocarb^32^. Resistance to bendiocarb has also been reported in other *An. funestus* isolates from DRC collected in Tchonka^14^. The original study in Southern Africa only found heterozygous (R/S) genotypes, but in Malawi homozygous (R/R) were also detected, demonstrating higher resistance to bendiocarb than R/S genotypes^60^. In this study, the resistant allele appeared in 14.7% of samples, with only one sample classified as R/R. Since there has never been widespread use of carbamates or organophosphates in the DRC, it is possible that the N643I mutation is playing another role in resistance/cross-resistance to other insecticides or imparts a fitness advantage.

In the *CYP6P4* gene, the I288N, G289R, N291S/T, L294V, K295E, and E297K SNPs all occur within the variable region of the protein, close to I236M, which is a mutation linked to pyrethroid resistance in *An. gambiae* s.s. and *An. coluzzii*^37^. Some of these SNPs appear in a block of high linkage, probably due to close proximity. Other genetic variants were detected in this gene, including in codon 414, where an isoleucine changes to a leucine. This change is unlikely to result in resistance to insecticides in *An. funestus*, as leucine is the reference amino acid for both *An. gambiae* s.s. and *An. arabiensis*. The other detected substitution D404N occurs in the conserved amino acid region, but is not proximal to the catalytic sites, so probably not involved in resistance. Our *CYP6P4* amplicon was designed based on the identification of deltamethrin binding site described previously, and believed to bind around the Pro^376^, Leu^380^, and Ser^381^^61^. However, additional, or modified amplicons can be included for *CYP6P4* if other positions are found to be important.

Of the three missense SNPs (G80A, K146T, V134M) found in the *GSTe2* amplicon, G80A is unlikely to have an impact on resistance, as other *Anopheles* spp. such as *An. sinensis* and *An. atroparvus* have alanine as the reference amino acid at this position. Similarly, the K146T alteration is present in other *Anopheles* spp. that have threonine as the reference amino acid at this position. For the V134M mutation, this position appears highly conserved across *Anopheles* spp. Codon position 134 exists within the H5 helix in the *GSTe2* protein, however H5 does not appear to play a part in DDT binding to *GSTe2*^35^. Mutations at nearby codon positions 131 and 139 have been previously reported and are not believed to alter insecticide susceptibility^35,36^. V134 was identified as a highly replaceable site across the GST family.

The five non-synonymous mutations found in the *vgsc* gene (F763L, I768L/M, L788F, and G793C) occur in the IIS1 domain of the VGSC protein. A T791M mutation has been previously reported in this region in *An. gambiae*, but no association with insecticide resistance was established^62^. In our work, the F763L, I768L, and G793C mutations all result in changes to amino acids found in other species at that position. The I768M and L788F mutations have not been observed in other *Anopheles* spp. For the I768M mutation, there was variation observed between species at codon 768, but methionine was not present. Whilst the leucine at codon 788 was highly conserved across species with no phenylalanine being reported previously. Future studies involving the analysis of genotype–phenotype associations in *An. funestus* populations could identify the possible involvement of these SNPs in insecticide resistance.

The absence of previously reported *vgsc-kdr* mutations and the *ace-1* G119S SNP^27,30^ is not unexpected, as these have not yet been observed in *An. funestus* populations. Other molecular mechanisms are involved in resistance to pyrethroids in this vector species. However, the continued attempts to detect the classic *kdr* mutations in *An. funestus* are necessary due the speed this highly favourable polymorphism can spread through the population, as seen with *An. gambiae*^27,63^*.* In DRC, *An. funestus* populations resistant to pyrethroid have been reported, likely due to the use of pyrethroid-only LLINs in the country^64^. It is therefore essential to investigate the genetic variants involved in insecticide resistance in *An. funestus*, due to the speed with which some of these highly favourable polymorphisms can spread through a population, as observed previously with *An. gambiae*^27,63^.

The cytochrome P450 genes were included in our panel because of their association with metabolic-based insecticide resistance. The previously described 2bp insertion within the *CYP6P9a* promoter region was detected in > 90% of our samples. This frequency is consistent with estimates based on applying a restriction fragment length polymorphism (RFLP) *CYP6P9a* diagnostic assay to a cohort of Tchonka and Tushunguti samples (82–98%)^10,39^. The 2bp insertion has been identified as a potential marker for pyrethroid resistance, being tightly linked with a resistant phenotype^39^. Other cytochrome P450 genes, such as *CYP6P9b*, have also been involved in insecticide resistant phenotype, particularly associated with elevated gene expression^65^. These loci can be integrated in the amplicon assay, particularly when genetic markers in these genes are uncovered to be involved in the resistance phenotype.

Of the phylogenetic markers included in the amplicon panel, those in *ITS2* showed the least utility for investigations into genetic diversity or relatedness. In contrast, the mitochondrial genes, *cox-1* and *mt-ND5*, showed more promise for speciation and population delineation. The *cox-1* gene was able to identify four samples that had been misclassified as *An. funestus*. Visual identification of mosquito species requires skilled and experienced individuals, but such identification can often be of limited use due to sample degradation. The non-*An. funestus* isolates were found to be *An. gambiae* and *An. coustani*, both known vectors in the region. *An. gambiae* is the focus of many vector control strategies across Sub-Saharan Africa, due to its large contribution to malaria transmission. *Anopheles coustani* is considered a secondary malaria vector across Central and Southern Africa. It is highly zoophilic and endophilic, differs sufficiently enough in behaviour to avoid many traditional vector control methods, and therefore its capacity for transmitting malaria is beginning to be taken more seriously^66,67^. Also such ability to escape IRS and LLINs through its behaviour, might be the origin of a future epidemic resurgence of malaria after the main malaria vector An. *gambiae* has been controlled. Whilst the occurrence of these species in this study could be the result of incorrect morphological identification, it may also be an example of species introgression^68^. BLAST analysis of the *cox-1* sequences for these misclassified samples revealed a 91–94% identity to *An. funestus,* compared to the 97–98% identity to the other species. Introgression of genes in *An. funestus* has previously been reported, but to confirm it is occurring here would require whole genome sequencing combined with a comparative genomic analysis^69^.

For both mitochondrial genes, high haplotype diversity was observed in the context of the very low nucleotide diversity. This suggests a high number of low frequency variants, which has been observed for other *Anopheles* spp.^43^. The smaller sample size tested here may contribute to the low frequencies observed, so increasing the number of specimens screened with this amplicon panel would provide greater insights into the population dynamics.

Our study has demonstrated the utility of an amp-seq panel as a viable screening technique for SNPs associated with insecticide resistance. The detection of previously unreported missense SNPs also demonstrates its potential usage for the identification of new SNPs that may be involved in insecticide resistance, if used in tandem with phenotypic studies. Currently the use of this panel in a field setting may be limited by access to sequencing platforms, and a lack of bioinformatics expertise, and as such could be of more use in a research setting. However the use of a portable sequencer such as the long-read MinION could help to overcome this, along with a graphical web interface platform for data analysis, as successfully implemented for malaria and tuberculosis^70,71^.

Importantly, informed vector control methods are needed to meet the World Health Organization goals of reducing malaria mortality by 90% within the next 7 years. Whilst gains have been made since this target was established, in recent years the number of cases has stabilised. New impetus is needed for large-scale surveillance studies with high throughput molecular tools to rapidly inform policy choices and reduce malaria cases. Our assay, which can be easily extended to other loci, represents a tool and opportunity to perform molecular surveillance in a vector heavily involved in malaria transmission across Africa.

### Supplementary Information

Supplementary Information.

## Data Availability

All raw sequence data is listed in the European Nucleotide Archive (Project ID: PRJEB61194, Accession numbers: ERR11507573–ERR11507628.
