# Supplementary material for: Detection of insecticide resistance markers in Anopheles funestus from the Democratic Republic of the Congo using a targeted amplicon sequencing panel
Source: Sci Rep. 2023 Oct 13;13:17363. doi: 10.1038/s41598-023-44457-0 (PMC10575962; doi:10.1038/s41598-023-44457-0)
Supplement: Supplementary file 1 — Supplementary Information. [file 41598_2023_44457_MOESM1_ESM.docx]

**Supplementary table 1.** Amplicon targets, primers sequences, and size (bp)

| ***Anopheles funestus* Primers information** | | | | | | | |
| --- | --- | --- | --- | --- | --- | --- | --- |
|  | Target Gene | Amplicon | Accession ID | Target SNP | Forward primer | Reverse Primer | Product Size (bp) |
| Insecticide  Resistance | *vgsc* | VGSCIa | AFUN000494 | n/a | TGCTTGGTTAAACCGAATAAAA | GCTTCGAACAATCGAAATCA | 479 |
|  |  | VGSCIb |  | V410L^22^ | TTTTCTTCGATGGTGTGCAA | ATGTGGTTGGCATTGTTTCA | 439 |
|  |  | VGSCIIa |  | n/a | AATGCTCGCCTACGATGTTC | CGTTGCTGTGAAGAACTGGA | 463 |
|  |  | VGSCIIb |  | L1014F/S^19,20^ | AGTGCTGGTGAACGCCTAAT | CATTTGCTATGTTCGCCTTG | 484 |
|  |  | VGSCIII |  | F1534C^24^/ N1575Y^23^ | GCGACATTCAAAGGATGGAT | GAAGCCGATGAACAACATGA | 513 |
|  |  | VGSCIV |  | D1763Y^21^ | TGTACTGCGTTTGGTGAAGG | CTTCCGTTGCTTGGGAATAG | 528 |
|  | *ace-1* | ACE1_Ia | AFUN011616 | n/a | CATCACTACCACCACCACCA | GCCAAGCCACACATCTACCT | 500 |
|  |  | ACE1_Ib | AFUN011616 | G119S^25^ | GAGCGGCAAAAAGGTAGATG | GCTGATAGCACACCTCCACA | 495 |
|  |  | ACE1_II | AFUN011616 | N485I^32^ | GCTACACCGAGGACGAGAAA | CTAGCAGCACAACCAGATCA | 480 |
|  | *GSTe2* | GSTe2 | AFUN015809 | L119F^30,31^ | CCTTCTGGCTGGTGATCATT | ACCCAGCAACAAAGTCATCC | 496 |
|  | *rdl* | RDL1 | AFUN016339 | A296S/ V371I^33,34^ | AACTCGCCCACAATGAAAAA | TTTAATGGGCAAACGGAAAG | 488 |
|  |  | RDL2 | AFUN016339 | T345S^34^ | AACACTTGTGATGCTTTTCGT | GAACCGTACCTCCTGGAACA | 482 |
|  | P450 (*CYP6P9a*) | CYP6P9a* | AFUN015792 | aa insertion^39^ | AGGTTATTTGGACGAACGTGA | CAAAACAAGTTAAGTGCCTGTAAGA | 496 |
|  | P450 (*CYP6P4*) | CYP6P4 | EU852651.1 | P376, L380, & S381^62^ | AGGTTATTTGGACGAACGTGA | CTGGGTAAAACTCGGGATCA | 496 |
| Phylogeny | Mitochondria  *(cox-1 & ND5)* | COX-1 | AF15KAPO-006 | n/a | GAGCCCCTGATATAGCTTTCC | AAACTTCTGGGTGTCCAAAAA | 468 |
|  |  | Mt-ND5 |  | n/a | TCTTTTGTTTTATTGATTTCATCG | TCCTGCTGTAACCAAAGTTGAA | 496 |
|  | *ITS2* | ITS2 | MK129243.1 | n/a | TCTAGTGTCGTGGGGGAAAC | AGCACGTTGTCCGAATATCA | 498 |

*These primers were designed by Weedall et al., 2019^38^

**Supplementary Table 2.** Average coverage, and number of variants identified for each amplicon

| Amplicon | Coverage | SNPs | NS SNPs | INDELs |
| --- | --- | --- | --- | --- |
| All | - | 351 | 18 | 26 |
| ACE1_I | 193.19 | 15 | 0 | 2 |
| ACE1_II | 722.50 | 11 | 0 | 0 |
| ACE1_III | 671.56 | 23 | 1 | 0 |
| COI | 2194.28 | 64 | 0 | 1 |
| CYP9P4 | 574.96 | 35 | 9 | 0 |
| CYP9P6a | 938.42 | 55 | 0 | 6 |
| GSTe2 | 846.42 | 18 | 3 | 2 |
| ITS2 | 3684.57 | 6 | 0 | 0 |
| mt-ND5 | 1059.01 | 13 | 0 | 0 |
| VGSCIa | 1205.03 | 5 | 0 | 2 |
| VGSCIb | 1012.69 | 5 | 0 | 2 |
| VGSCIIa | 2521.66 | 39 | 5 | 2 |
| VGSCIIb | 839.73 | 7 | 0 | 0 |
| VGSCIII | 870.34 | 12 | 0 | 0 |
| VGSCIV | 512.47 | 8 | 0 | 0 |
| Rdl1 | 1326.72 | 7 | 0 | 6 |
| Rdl2 | 843.04 | 18 | 0 | 3 |

a)

D404N

E297K

K295E

L294V

N291S

N291T

G289R

I288N

I414L

b)

F763L

I768L

G793C

I768M

L788F

**Supplementary figure 1.**  Linkage disequilibrium networks for non-synonymous SNPs detected in a) CYP6P4, and b) VGSC (domain II) amplicons. An r^2^ value of 0 indicates no linkage, whereas a value of 1 suggests strong linkage. Figures created using vcftools and R package Gaston.

**Supplementary Figure 2.** Maximum-likelihood tree constructed using ITS2 gene sequences generated in this study (n=77), alongside other publicly available *An. funestus* ITS2 sequences (n=36), (Cameroon = 5, Ethiopia = 1, Kenya = 1, Madagascar = 2, Malawi = 3, Mozambique = 8, Zambia = 16). The tree was built using the maximum-likelihood method assuming GTR model of nucleotide substitution, with the gamma model of heterogeneity rate.
